# Supplementary figures and images for: Deep-learning-based three-dimensional label-free tracking and analysis of immunological synapses of CAR-T cells
Source: eLife. 2020 Dec 17;9:e49023. doi: 10.7554/eLife.49023 (PMC7817186; doi:10.7554/eLife.49023)

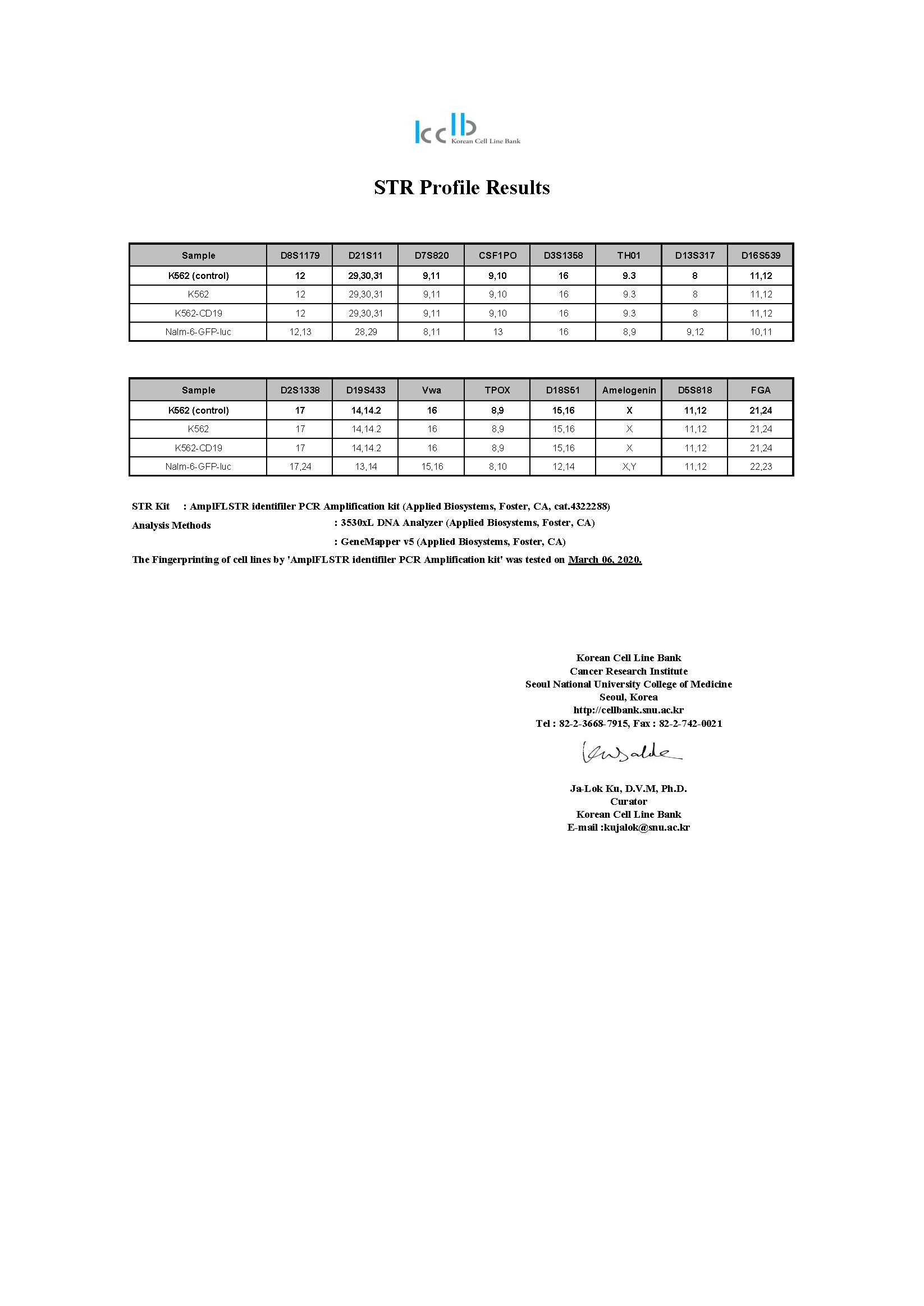

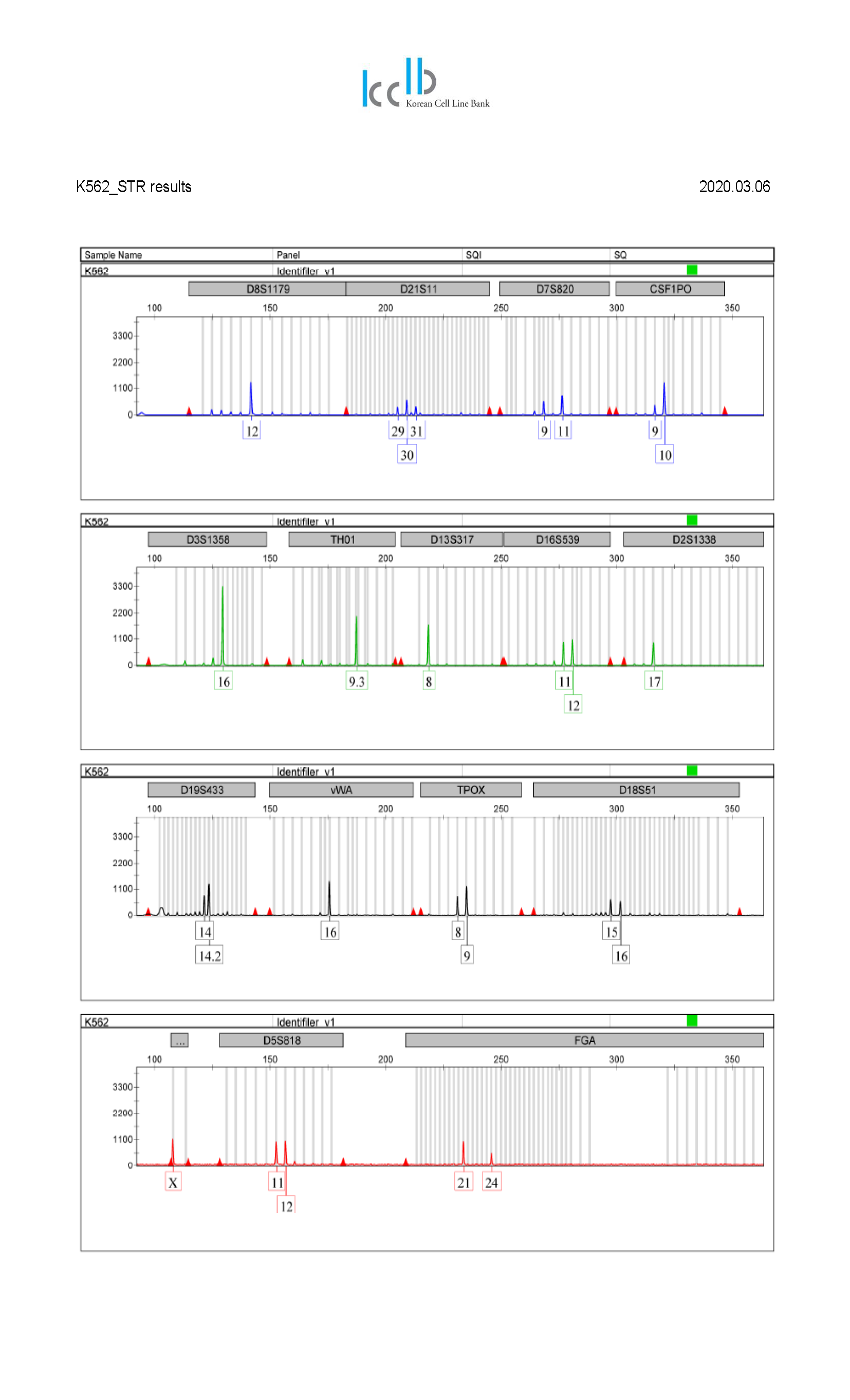

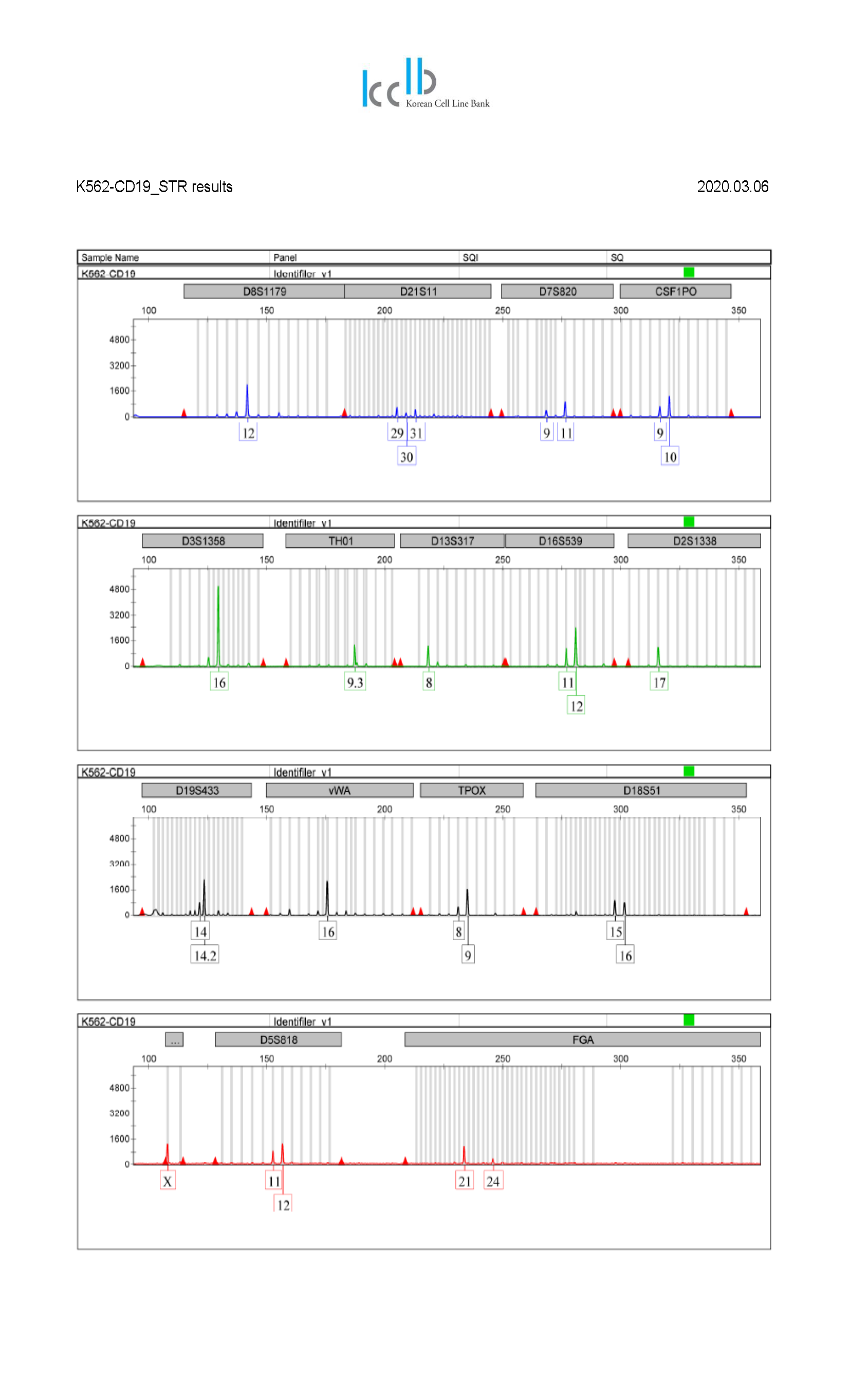

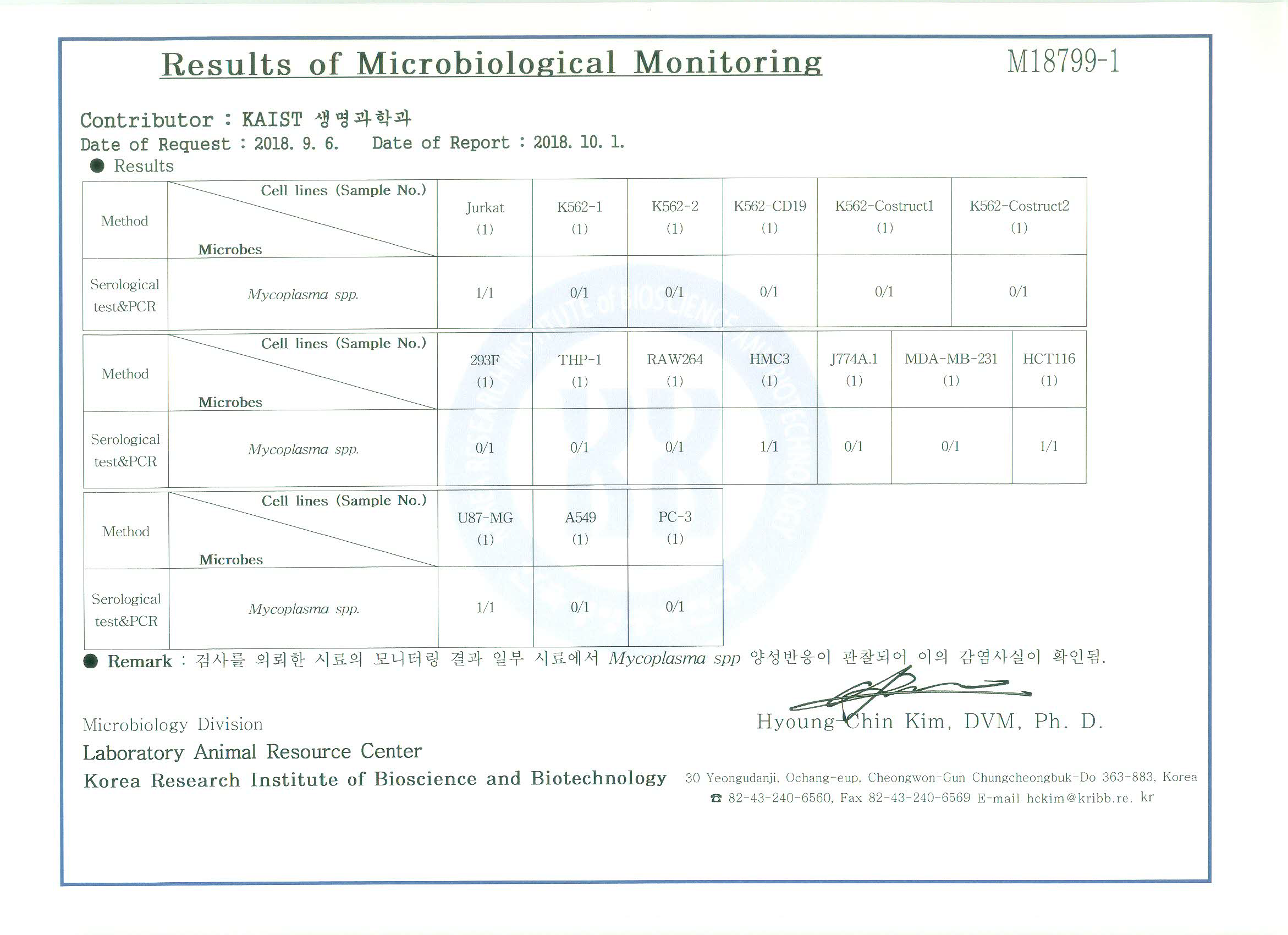

Supplement: Supplementary file 1. [file elife-49023-supp1.docx]
